# Application Modes Affect Two Universal Adhesive Systems' Nanoleakage Expression and Shear Bond Strength

**DOI:** 10.1155/2021/7375779

**Published:** 2021-09-30

**Authors:** A. S. Bakry, M. A. Abbassy

**Affiliations:** ^1^Restorative Dentistry Department, Faculty of Dentistry, King Abdulaziz University, Jeddah, Saudi Arabia; ^2^Conservative Dentistry Department, Faculty of Dentistry, Alexandria University, Alexandria, Egypt; ^3^King Fahd Medical Research Center, King Abdulaziz University, Jeddah, Saudi Arabia; ^4^Department of Orthodontics, Faculty of Dentistry, King Abdulaziz University, Jeddah, Saudi Arabia; ^5^Alexandria University, Alexandria, Egypt

## Abstract

**Objectives:**

The aim of this study was to evaluate the shear bond strength and the nanoleakage expression of CLEARFIL Universal Bond Quick and Tetric N-Bond adhesive systems bonded to dentin.

**Materials and Methods:**

100 freshly extracted human premolar teeth were utilized. The teeth were sectioned to expose dentin. All dentin specimens were assigned into 4 experimental groups; 2 groups had Universal Bond Quick (Universal_self_ group) and Tetric N-Bond (Tetric_self_ group) applied in the self-etch mode, while 2 groups had Universal Bond Quick (Universal_total_ group) and Tetric N-Bond (Tetric_total_ group) applied in the total-etch mode. *n* = 15 for shear bond strength and *n* = 10 for nanoleakage experiment. One-way ANOVA and Kruskal-Wallis test were utilized to analyze the shear bond strength test and the nanoleakage expression, respectively.

**Results:**

The highest significant bond strength value was recorded by the Tetric_self_ specimens (*p* < 0.05) when compared to the remaining three groups. There were no statistically significant differences between the shear bond strength values recorded in the Tetric_total_, Universal_self_, and Universal_total_ groups (*p* < 0.05). Both bonding systems applied in the self-etch mode (Universal_self_, Tetric_self_) had no silver nitrate deposits in the hybrid layer and the hybrid layer-adhesive interface (*p* < 0.001); however, both bonding systems applied in the total-etch mode (Universal_total_, Tetric_total_) had silver nitrate deposits in the hybrid layer, the hybrid layer-adhesive interface, and the bonding layer (*p* < 0.001).

**Conclusion:**

Applying the Universal Bond Quick and Tetric N-Bond in the self-etch mode exhibited better results in terms of nanoleakage expression. Universal Bond Quick showed the stability of the shear bond strength to dentin when applied using the total-etch or self-etch modes. Tetric N-Bond showed significant deterioration in bond strength when applied in the total-etch mode and exhibited the highest bond strength when applied in the self-etch mode.

## 1. Introduction

The introduction of self-etch adhesives in the late 1980s revolutionized the science of dental adhesives; many advantages were associated with the use of the self-etch approach to the dentin surface which may include its ease of use [1] (by decreasing the number of steps needed for the dentin bonding procedures) and less technique sensitivity [1] (by not adopting the wet bonding technique utilized with the total-etch approach).

Moreover, the less aggressive acidic treatment associated with the self-etch approach (compared to the total-etch approach) preserved the smear plugs into the dentinal tubules thus decreasing the deteriorating effect of the pulpal pressure on the adhesive-dentin interface [2, 3].

One of the unique features associated with the use of self-etch adhesives possessed is its capability to simultaneously condition and penetrate the dentin collagen network [1, 4, 5]. This capability improved the adhesives encapsulation for most of the conditioned exposed dentin collagen, thus increasing the sealing properties of the dentin-adhesive interface [1, 4, 5].

Application of the self-etch adhesives on dentin cervical cavosurface margins in Class V cavities rendered these margins resistant to a simulated caries attack when compared to etching these margins with phosphoric acid prior to bonding [6].

All of these advantages were reflected as a clinical success of the mild self-etch adhesives in the form of less postoperative pain symptoms and prolonged clinical performance for most of the 2-step mild self-etch systems [1, 4]. This prolonged clinical performance decreased the demand for replacing the restorations due to recurrent caries and thus preserve the integrity of the remaining tooth structures and comply with the minimal intervention concepts that advocate the preservation of tooth structures [7].

The new generations of one-step self-etch adhesives are currently called universal dental adhesives due to their unique feature of durable bonding to coronal dental tissues [8–11] and different restorative materials (after proper treatment of the filling material surfaces) [8–11]. This unique feature might be attributed to the incorporation of 10 MDP adhesive monomer in their composition [8–12]. Previous research showed that 10 MDP is capable of bonding to titanium, ceramics, and metallic alloys [8–11]. However, manufacturers claim that these systems have the capability of bonding to dentin either in a total-etch (etching of dentin with phosphoric acid) or in a self-etch mode. The current study shed the light on the different properties of the dentin-adhesive interface when two one-step self-etch adhesive systems were applied either in the total-etch mode or in the self-etch mode. The null hypothesis is that the modes of adhesive applications will not affect the shear bond strength and the nanoleakage expression at the adhesive-dentin interface.

## 2. Materials and Methods

### 2.1. Selection and Preparation of Teeth

100 premolar teeth freshly extracted for orthodontic purposes were utilized in the current study. The teeth were collected from the oral surgery department after obtaining the permission of the ethical committee of the faculty. The teeth were hand-scaled from any calculus or soft tissues. The teeth were stored in 0.1% thymol till the start of the experiment according to the guidelines approved by the University. The number of specimens assigned to each group was adopted according to the threshold for significance which was set at 0.05 and means and standard deviation obtained in a pilot study and the power of test which was set at 80% [13]. Randomization of the specimens was done using a computer program (Excel 2007, Microsoft, Redmond, WA, USA). All teeth were examined by a light microscope to exclude any teeth having cracks, restorations, demineralization, or any defects. Intra- and interexaminer calibrations were conducted before the actual recording of the obtained results. The teeth were sectioned using a low-speed diamond saw (Isomet, Beuhler, IL, USA) under water cooling conditions exposing the midcoronal dentin. All specimens were ground using 600 grit SiC paper under wet conditions to create a standardized smear layer [14].

### 2.2. Experimental Design

All dentin specimens were assigned into 4 experimental groups; 2 groups had Universal Bond Quick (Universal_self_ group) and Tetric N-Bond (Tetric_self_ group) applied in the self-etch mode, while 2 groups had Universal Bond Quick (Universal_total_ group) and Tetric N-Bond (Tetric_total_ group) applied in the total-etch mode. *n* = 15 for shear bond strength and *n* = 10 for nanoleakage experiment.

### 2.3. Shear Bond Strength

60 samples were used in the current experiment. 15 treated dentin samples were selected from each group (Table 1). Each treated dentin specimen had a Teflon tube having 3 mm and an internal diameter of 3 mm fixed onto the dentin surface, and Tetric N Ceram composite (Ivoclar Vivadent AG, Schaan, Liechtenstein) was condensed into it. The adhesive system and the composite resin were light cured by a light curing unit for 10 s and 30 s, respectively. All specimens were stored in distilled water for 24 hours in an incubator having a temperature set at 37°C. The specimens were mounted on the universal testing machine (ElectroPlus E1000, Instron, Canton, MA, USA) and subjected to a shear force at the composite resin-dentin at a crosshead speed of 0.5 mm/min.

### 2.4. Nanoleakage Expression Experiment

**Figure 1:**
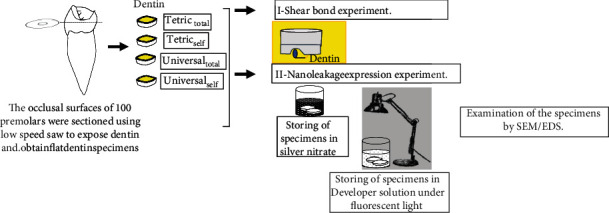
Summary for the experimental procedures.

30 specimens were utilized in this experiment. 10 teeth were randomly selected from each group. The selected specimens were vertically cross-sectioned with a diamond saw under water cooling through the dentin-resin interface. The bonded slabs were ground and polished using #1000 silicone carbide paper under wet conditions. The specimens were dried, then coated with two layers of fast-drying nail varnish applied up to 1 mm from the bonded interface. The specimens were exposed to ammoniacal silver nitrate in total darkness for 18 h, rinsed thoroughly, and immersed in photodeveloping solution (Kodak, NY, USA) for 6 h under fluorescent light to reduce silver ions into metallic silver [15]. The silver-stained resin-bonded specimens were lightly polished to remove the superficial silver remnants [2, 5, 15, 16], followed by drying the specimens and preparing it for SEM observation. The specimens were examined using SEM/EDS (JCM-6000, NeoScope, JEOL, Tokyo, Japan), and line scans were examined across the resin-dentin interface [2, 5, 15]. The nanoleakage expression was observed and recorded in three areas: hybrid layer, hybrid layer-adhesive interface, and adhesive layer. All of the experimental steps are summarized in Figure 1.

### 2.5. Statistical Analysis

One-way ANOVA was used to analyze the shear bond strength, whenever there was a significant difference, the post hoc test was utilized for further analysis. The nanoleakage expression results were analyzed the by Kruskal-Wallis test [14]. The level of significance for all tests was set at *α* = 0.05. Software utilized for analysis was SPSS v24 (IBM, Armonk, US).

## 3. Results

### 3.1. Shear Bond Strength

Table 2 presents the means and standard deviations for the shear bond strength of dentin specimens. The results show that the application mode affected the Tetric N-Bond system (*p* < 0.001) while it did not affect the Universal Bond Quick system (*p* < 0.05). The highest significant bond strength value was recorded by the Tetric_self_ specimens (*p* < 0.05) when compared to the remaining three groups. There were no statistically significant differences between the shear bond strength values recorded in the Tetric_total_, Universal_self_, and Universal_total_ groups (*p* < 0.05).

### 3.2. Nanoleakage Expression Experiment

**Figure 2:**
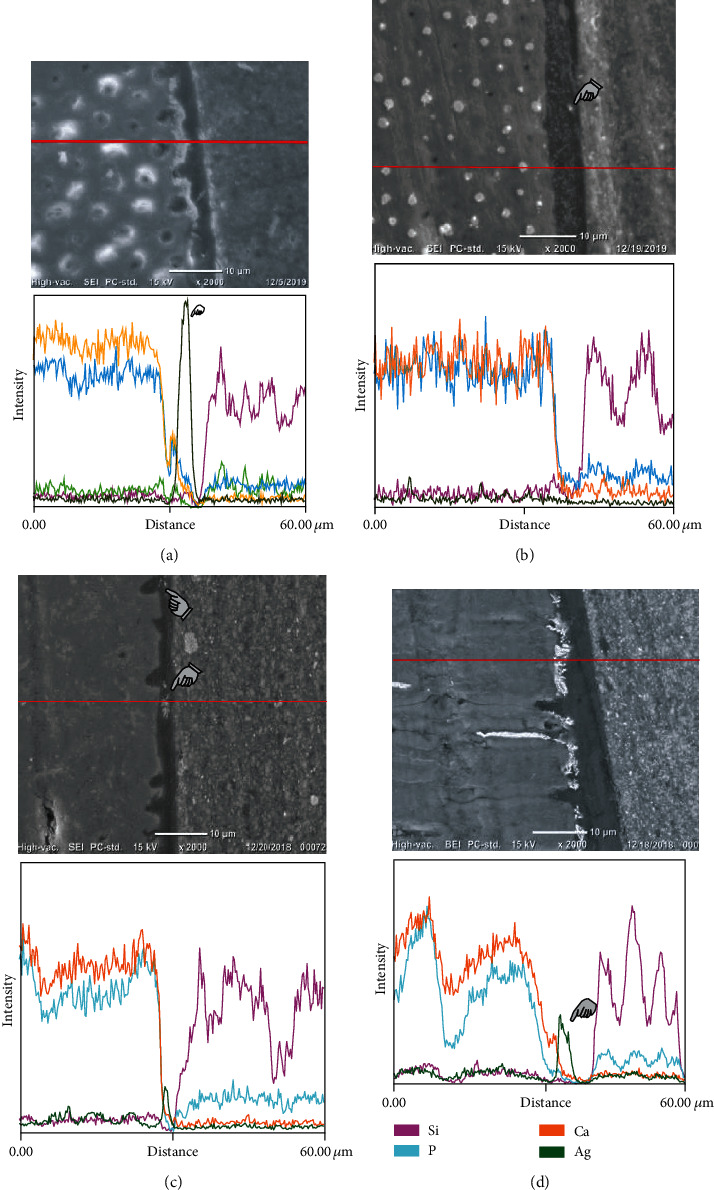
SEM/EDS analysis for EDS line scans across the interface between dentin and the adhesive system. (a) Tetric_total_ group representative sample showing heavy infiltration of silver nitrate deposits within the hybrid layer, hybrid layer-adhesive interface, and bonding layer itself. Finger pointer showing diagnostic peak of silver. (b) Tetric_self_ group representative sample having finger pointer demarcating sporadic silver nitrate particles detected in the bonding layer. (c) Universal_self_ group representative sample having finger pointers demarcating sporadic silver nitrate particles. (d) Universal_total_ group representative sample showing heavy infiltration of silver nitrate deposits within the hybrid layer, hybrid layer-adhesive interface, and bonding layer itself. Finger pointer showing the diagnostic peak of silver.

Figure 2 and Table 3 refer to the nanoleakage expression observed in the 4 groups. The mode of application significantly affected the nanoleakage expression (*p* < 0.001). Both bonding systems applied in the self-etch mode (Universal_self_, Tetric_self_) had no silver nitrate deposits in the hybrid layer and the hybrid layer-adhesive interface (*p* < 0.001); however, sporadic particles of silver nitrate particles were observed in the bonding layer itself. Both bonding systems applied in the total-etch mode (Universal_total_, Tetric_total_) had silver nitrate deposits in the hybrid layer, the hybrid layer-adhesive interface, and the bonding layer (*p* < 0.001).

## 4. Discussion

The null hypothesis in the current experiment was rejected as the mode of adhesive system application affected the nanoleakage expression and had significant effect on the shear bond strength. The limitations of this study might include the utilization of the shear bond strength test with cylindrical-shaped composite cylinders having a diameter of 3 mm. If we have utilized the microtensile bond strength test, the tested bonded surface would have been smaller [17] and thus would be expected to contain less dentinal defects (e.g., cracks) and would have yielded higher bond strength values [17]. However, a previous study [18] comparing the reliability of both tests highlighted that despite the low bond strength values, the shear bond strength test is capable of reliable and reproducible results and that it is a suitable method for comparing the bond strength of various dental adhesive systems [18].

Regarding the other tests adopted in the current experiment, it is worth mentioning here that the nanoleakage test is considered an important index for the durability and the sealing ability of adhesive restorations [5, 14–16]. In the current experiment, nanoleakage expression was observed in the interfaces of the adhesive system applied with the total-etch mode which may be linked to several mechanisms; among these, it may be suggested that etching dentin by phosphoric might have caused complete denudation of the collagen fibrils from their hydroxyapatite coating leading to the collapse of the collagen network with subsequent hindering of the adhesive monomer from infiltrating the collagen network. The amount of water contained in both adhesive systems and not employing the moist bonding technique (as recommended by the manufacturers) seemed to be unsuccessful in preventing the collagen network from collapse.

The presence of collagen fibrils which are not encapsulated by a resin coating after polymerization of the adhesive resin and forming the hybrid layer may lead to a sequence of detrimental effects on the adhesive restorations [19–21]. These effects may include activation of the MMPEs present in the collagen structures leading to their degradation and possible infiltration of the oral fluids and bacteria in this critical zone and eventual failure of the dentin-resin interface [19–21]. One of the most sensitive components whose stability will be compromised by the deteriorating sealing ability of the resin-dentin interface is the hydroxy ethyl methacrylate (HEMA) leading to the accelerated degradation of the adhesive resin itself over time [4, 22].

**Figure 3:**
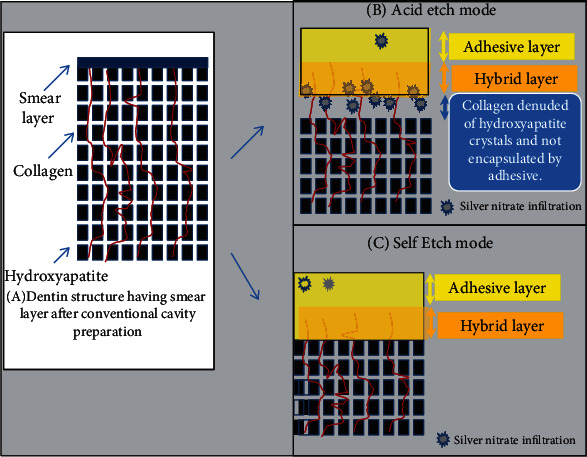
(a) Dentin structure after conventional cavity preparation using high-speed contra-angle handpiece (please note the formation of the hybrid layer). (b) After acid etching of the dentin, a poorly sealed area of collagen denuded from its hydroxyapatite crystal coating was created that was infiltrated by silver nitrate particles. (c) Encapsulation of the collagen by resin forming a good, sealed hybrid layer interface. Sporadic silver nitrate particles can be eventually seen in the adhesive layer of all adhesive systems in the self-etch mode due to improper evaporation of remaining moisture contained within the adhesive structure.

On the other hand, employing the same bonding systems without employing the phosphoric acid etching procedure exhibited less nanoleakage expression which may be attributed to the simultaneous conditioning and penetration of the tested adhesive systems within the dentin structure [1, 4] which might have avoided the collapse of the collagen fibrils as was previously explained. The observation of sporadic silver nitrate deposits in the adhesive layer when applying both bonding systems in both modes may be attributed to the chemical formula complexity of the tested bonding system that may suffer from phase separation [23] and improper evaporation of the incorporated solvents and residual water content within the adhesives utilized [23–25] which might have caused the reported sporadic silver nitrate particle dispersion in the adhesive layer. A summary of the nanoleakage expression results and underlying mechanism of occurrence obtained in the current experiment is presented in Figure 3.

On the other hand, the mode of applying the adhesive systems did not affect the shear bond strength of Universal Bond Quick values which may be attributed to the multifunctional hydrophilic amide monomer contained in Universal Bond Quick that has a lower octanol/water partition coefficient; logPow (−0.7) than HEMA (logPow (0.3) [26], indicating more hydrophilicity before polymerization which might have relatively assisted the penetration of the bonding system in the Universal_total_ group which was expressed by the relatively weak EDS silver peak observed in the Universal_total_ group when compared to the strong silver peak observed in the Tetric_total_ group (Figure 2). Although this hydrophilic amide monomer exhibits hydrophilic nature before polymerization, it shows minimal water sorption [26] and good mechanical properties when polymerized in wet conditions [26], which may be attributed to the ability of this monomer to form a three-dimensional polymer network upon light polymerization that renders it less prone to moisture contamination. The good mechanical properties of the hydrophilic monomer might have reinforced the mechanical properties of the hybrid layer in the Universal_self_ group and caused its SBS stability when compared to the Universal_total_ group.

On the contrary, Tetric N-Bond showed significant deterioration in its bond strength when applied in the total-etch mode which may be attributed to the high content of HEMA in this adhesive system which is 10-25% compared to the Universal Bond Quick that contains approximately 2.5%. It is speculated that HEMA in Tetric N-Bond was much affected by the fluids seeping into the denuded collagen fibrils of the hybrid layer that was created due to applying the phosphoric acid in the Tetric_total_ group. This dentin-adhesive interface contamination by water might have led to HEMA water sorption [27] and a decrease in the mechanical properties of Tetric N-Bond that was manifested by the reported decrease in shear bond strength observed in the current experiment.

Interestingly, the high content of HEMA seemed to improve the performance of the Tetric N-Bond when applied in the self-etch mode by improving the penetration of the bonding system into the self-etched [27] dentin structure which was manifested by recording the highest bond strength values among the tested groups.

Previous literature showed controversial outcomes when compared to our results. On the one hand, a clinical study [28] compared the clinical performance of various adhesives in the total-etch versus self-etch mode and found no significant differences; however, the selection criteria for the cases included excellent oral hygiene patients with noncaries lesions involving superficial layers of dentin free from hypersensitivity. These specific criteria may not represent most of the clinical cases that necessitate restorations because noncaries lesion cases not suffering from hypersensitivity symptoms might have different chemical and mechanical features from caries-affected dentin forming the floor and part of the walls of most prepared cavities [29, 30].

On the other hand, another clinical study [31] showed that a universal bonding system applied on dentin in the self-etch mode prior to the restorative phase yielded acceptable results after 24 months of follow-up [31]. Moreover, our result agreed with a previous study that recommended the use of universal adhesives in the self-etch mode rather than the total-etch mode [32].

Additionally, a previous review of literature [33] emphasized the role of the nanoleakage expression in the degradation of the resin-dentin interface and highlighted the role of the chemical composition of universal bonding systems in expressing nanoleakage at the dentin-resin interface [33].

The outcome of this in vitro study should be interpreted carefully in terms of its clinical relevance because this study did not include any durability challenges such as pH cycling, thermal cycling, and cyclic loading which simulate the physiological conditions inside the oral cavity that include repeated expansion and contraction stresses due to oral cavity temperature variation and erosive challenges induced by acidic chemicals in saliva and food [33, 34]. Thus, it may be speculated that the aforementioned factors may enhance the nanoleakage expression at the adhesive-dentin interface in the clinical situation.

## 5. Conclusion

Applying the Universal Bond Quick and Tetric N-Bond in the self-etch mode exhibited better results in terms of nanoleakage expression. Universal Bond Quick showed the stability of the shear bond strength to dentin when applied using total-etch or self-etch modes. Tetric N-Bond showed significant deterioration in bond strength when applied in the total-etch mode and exhibited the highest bond strength when applied in the self-etch mode.

## Figures and Tables

**Table 1:** Chemical composition and application mode of the tested materials.

Material	Composition	pH	Grouping and application mode
Universal Bond Quick (Kuraray Noritake, Tokyo, Japan)	BisGMA, HEMA, ethanol, 10 MDP. Hydrophilic aliphatic dimethacrylate, colloidal silica, CQ, silane coupling agent, accelerators, initiators, water	2.3	(Universal_self_) group (1) Apply bond with rubbing action. (2) Immediately dry for 5 seconds with oil-free gentle air stream. (3) Light cure for 10 s
			(Universal_total_) group (1) Apply etchant for 10 s. (2) Rinse. (3) Dry. (4) Apply adhesive as was described for the self-etch mode
Tetric Bond Universal (Ivoclar Vivadent, Schaan, Liechtenstein)	BisGMA (25-50%), water and ethanol (10-<25%), 2-hydroxyethyl methacrylate (HEMA) (10-<25%), phosphonic acid methacrylate (MDP) (10-<25%), diphenyl(2,4,6-trimethylbenzoyl)phosphine oxide (1-<2.5%), urethane dimethacrylate (0.3-<10)	2.5	(Tetric_self_) group (1) Apply bond with rubbing action for 20 s. (2) Dry for 5 seconds with oil-free gentle air stream. (3) Light cure for 10 s
			(Tetric_total_) group (1) Apply etchant for 10 s. (2) Rinse 10 s. (3) Dry 5 s. (4) Apply adhesive as was described for self-etch mode
Tetric N Ceram (Ivoclar Vivadent, Schaan, Liechtenstein)	BisGMA, Bis-EMA, TEGDMA, barium glass, ytterbium difluoride 56		Applied in all groups

**Table 2:** Microshear bond strength.

	Mean	SD	*N*
Tetric_self_	36.71^a^	4.87	15
Tetric_total_	22.79^b^	4.68	15
Universal_self_	26.1^b^	6.24	15
Universal_total_	25.59^b^	5.74	15

**Table 3:** Evaluation of nanoleakage location.

	Adhesive			Adhesive-hybrid layer interface			Hybrid layer		
	No	Slight	Distinct	No	Slight	Distinct	No	Slight	Distinct
Universal_self_ group	5	5	0	10	0	0	10	0	0
Universal_total_ group	5	5	0	0	0	10	0	0	10
Tetric_self_ group	7	3	0	10	0	0	10	0	0
Tetric_total_ group	5	5	0	0	0	10	0	0	10

## Data Availability

All data are available in the manuscript.
